# Histone deacetylase 6 inhibition rescues axonal transport impairments and prevents the neurotoxicity of HIV-1 envelope protein gp120

**DOI:** 10.1038/s41419-019-1920-7

**Published:** 2019-09-12

**Authors:** Erin D. Wenzel, Andrew Speidell, Sarah A. Flowers, Chengbiao Wu, Valeria Avdoshina, Italo Mocchetti

**Affiliations:** 1 Department of Pharmacology and Physiology, Washington, DC 20057 USA; 2 Department of Neuroscience, Georgetown University Medical Center, 3970 Reservoir Road NW, Washington, DC 20057 USA; 3 Department of Neurosciences, University of California, San Diego, 9500 Gilman Drive, La Jolla, CA 92093 USA

**Keywords:** Motor proteins, Cell death in the nervous system

## Abstract

Despite successful antiretroviral drug therapy, a subset of human immunodeficiency virus-1 (HIV)-positive individuals still display synaptodendritic simplifications and functional cognitive impairments referred to as HIV-associated neurocognitive disorders (HANDs). The neurological damage observed in HAND subjects can be experimentally reproduced by the HIV envelope protein gp120. However, the complete mechanism of gp120-mediated neurotoxicity is not entirely understood. Gp120 binds to neuronal microtubules and decreases the level of tubulin acetylation, suggesting that it may impair axonal transport. In this study, we utilized molecular and pharmacological approaches, in addition to microscopy, to examine the relationship between gp120-mediated tubulin deacetylation, axonal transport, and neuronal loss. Using primary rat cortical neurons, we show that gp120 decreases acetylation of tubulin and increases histone deacetylase 6 (HDAC6), a cytoplasmic enzyme that regulates tubulin deacetylation. We also demonstrate that the selective HDAC6 inhibitors tubacin and ACY-1215, which prevented gp120-mediated deacetylation of tubulin, inhibited the ability of gp120 to promote neurite shortening and cell death. We further observed by co-immunoprecipitation and confirmed with mass spectroscopy that exposure of neurons to gp120 decreases the association between tubulin and motor proteins, a well-established consequence of tubulin deacetylation. To assess the physiological consequences of this effect, we examined the axonal transport of brain-derived neurotrophic factor (BDNF). We report that gp120 decreases the velocity of BDNF transport, which was restored to baseline levels when neurons were exposed to HDAC6 inhibitors. Overall, our data suggest that gp120-mediated tubulin deacetylation causes impairment of axonal transport through alterations to the microtubule cytoskeleton.

## Introduction

A large proportion of the human immunodeficiency virus-1 (HIV)-positive population (50–70%) demonstrates cognitive impairment and memory dysfunction despite adherence to life-saving antiretroviral therapy (ART)^1,2^. These cognitive complications are known collectively as HIV-associated neurocognitive disorders (HANDs). Even in the post-ART era, patients with HAND have altered pathology in their brain and demonstrate subcortical white or gray matter loss when compared to non-infected patients^3^. Despite the inability of HIV to productively infect neurons^4^, HAND pathology consists of simplification of synapses and synaptodendritic injury^5^. Moreover, neuronal markers such as neurofilament light chain and Tau are increased in the cerebrospinal fluid or plasma of HAND subjects^6,7^, further suggesting neuronal injury. However, the sub-cellular mechanisms behind these neuropathological alterations are not fully understood.

HIV neuropathogenesis likely results from a interplay between host cellular events, including persistent neuroinflammation, impaired energy and lipid metabolism, drug use, and viral factors (reviewed in refs. ^1,8^). The envelope protein gp120, which is produced and shed during viral infection, appears to activate key mechanisms that could explain HIV-mediated loss of synapses^9,10^. Gp120 is endocytosed into neurons^11,12^ in a chemokine receptor, dynamin-dependent manner^13^, and once internalized, it binds to microtubules^14^. The association of gp120 with microtubules may play a role in gp120’s toxicity because displacing gp120 from microtubules prevents gp120-mediated loss of synapses as well as neuronal death^14^. Still, the mechanisms linking the gp120’s effect on microtubules and neuronal damage remain largely uncharacterized.

Microtubules are made of heterodimers of α- and β-tubulin, which, when assembled, undergo significant post-translational modifications including acetylation/deacetylation on lysine 40^15^. Deacetylation of tubulin occurs through the enzymatic activity of either histone deacetylase 6 (HDAC6) or sirtuin 2;^16,17^ however, several studies suggest that this deacetylation is primarily mediated by HDAC6^18,19^. Deacetylated tubulin causes microtubules to be vulnerable to degradation and more sensitive to microtubule-severing proteins^20^. Deacetylation of tubulin also impairs both anterograde and retrograde axonal transport^21,22^, intracellular functions essential for the survival of neurons. Because inhibiting tubulin deacetylation has proven to be efficacious in preventing neurodegeneration observed in animal models of Alzheimer’s and Huntington’s diseases^21,23^, characterizing the relationship between HIV, HIV proteins, tubulin deacetylation, and axonal transport may be therapeutically significant for HAND.

Axonal transport is mediated by motor proteins including kinesin-1 and dynein. These motor proteins carry essential cargo throughout the neuron in the anterograde and retrograde direction, respectively^24^. These include vesicles containing neurotransmitters or neurotrophic factors, including brain-derived neurotrophic factor (BDNF)^21,25^, and key organelles, such as mitochondria, to help regulate energetic demands of the neuron^26^. Previous work in our lab has shown that gp120 alters post-translational modifications on tubulin^27^, suggesting that this viral protein may alter axonal transport. Impaired axonal transport of BDNF is believed to initiate the neurodegenerative process observed in Huntington’s, Parkinson’s, and other neurodegenerative diseases^28,29^. These diseases, similar to HAND, are characterized by damage to the neuronal cytoskeleton. Thus, it is plausible to suggest that gp120 may cause synaptic simplification by impairing microtubule-mediated axonal transport^30^.

This study aimed to test whether HIV, through gp120, impairs the structure and function of the neuronal cytoskeleton. We report that gp120 decreases tubulin acetylation in neurons and disrupts axonal transport. Elucidating the mechanisms behind HIV-mediated synaptic simplification and neuronal dysfunction is essential in order to develop adjuvant therapies to prevent cognitive impairment.

## Materials and methods

### Reagents and antibodies

Gp120IIIB (cat#1001) and Tat (cat#1002) were purchased from ImmunoDX, LLC (Woburn, MA). Tubacin was purchased from Tocris (Minneapolis, MN) and Ricolinostat (ACY-1215) was purchased from Cayman Pharmaceuticals (Ann Arbor, MI). HIV-1 IIIB virus was obtained from Dr. Robert Gallo (cat#398)^31–33^ through the NIH AIDS Reagent Program, Division of AIDS, NIAID, NIH (Bethesda, MD).

### Animals

All experiments involving animals were performed in accordance with ethical standards and relevant guidelines for animal welfare outlined by the National Institutes of Health Guide for the Care and Use of Laboratory Animals and with approval from the Georgetown University Animal Care and Use Committee.

Adult male and female Sprague–Dawley rats (250 g, Charles River Inc., Germantown, MD) were anesthetized with ketamine/xylazine (80/10 mg/kg, intraperitoneally) and placed in a David Kopf stereotaxic frame (David Kopf Instruments, Tujunga, CA). Rats received an acute injection of vehicle control of 0.1% bovine serum albumin (BSA) in phosphate-buffered saline (PBS) unilaterally or 400 ng of gp120 in 0.1% BSA into the contralateral dorsal striatum (A/P+ 0.7, M/L+/− 3.0, and D/V −6.0 mm from the bregma) according to Paxinos and Watson^34^. Vehicle or gp120 was delivered over 10 min by a microperfusion pump at a constant rate of 0.2 µl/min for a total injection volume of 2 µl per hemisphere. After completion of each injection, the needle was left in place for an additional 5 min in order to accomplish quantitative diffusion of the volume delivered. Animals were returned to their cages after recovery. At the appropriate survival times, animals were deeply anesthetized, intracardially perfused with 0.1 M PBS (pH 7.4), and their whole brains removed for microdissection and analysis. No animals were excluded from analysis for any reason. Needle placement in the striatum was verified in all animals by the experimenter at the time of dissection. The hemisphere of gp120 injection was randomized and counterbalanced across animal sex and time point. For biochemical analysis via Western blot, investigators were blinded to side of injection until analysis was completed.

### Primary neuronal cultures

Primary rat cortical neurons were prepared from the cortex of embryonic (E17–18) Sprague–Dawley rats (Taconic, Derwood, MD, USA) as previously described^35^. Cells were plated onto poly-d-lysine pre-coated plates at a density of 25,000 cells/ml (immunocytochemistry), 200,000 cells/ml (transfection), or 500,000 cells/ml (all other experiments). Cells were grown on coverslips or CellBIND™ plastic (cat#08757214, Fisher Scientific, Pittsburgh, PA) for 14 days in vitro (DIV) at 37 °C in 5% CO_2_/95% air with one 50% media exchange on DIV7. Purity of neurons was routinely examined by microtubule-associated protein 2 (MAP2)-positive staining and contain between 95 and 98% neurons.

Primary astrocytes and microglia were prepared from Sprague–Dawley pups at postnatal days 1–2 according to an established protocol^35^. Purity was ensured by glial fibrillary acidic protein-positive staining. Purity of primary microglia was ensured by ionized calcium-binding adapter molecule 1-positive staining.

### Immunocytochemistry

After treatment, cells were rinsed gently with ice-cold 1× PBS and fixed with 4% paraformaldehyde/4% sucrose for 10 min at room temperature (RT). Cells were permeabilized with a 0.02% Triton X-100 solution in PBS for 10 min and then blocked with 1% BSA in PBS for 1 h at RT. Cells were incubated with an antibody against MAP2 (cat#M4403, 1:5000, MilliporeSigma, Burlington, MA) at 4 °C overnight. Corresponding goat anti-mouse secondary antibody AlexaFluor594 (cat#A11005, 1:1000, Thermo Fisher Scientific) was then incubated for 1 h at RT. After three washes with 1× PBS, coverslips were mounted with Fluoro-Gel with TES Buffer (Electron Microscopy Sciences, Hatfield, PA), and imaged with a Nikon (Tokyo, Japan) Eclipse microscope with a ×20 objective.

Live neurons in the microfluidic chamber were stained with 500 nM SiR-tubulin (cat#CY-SC002, Cytoskeleton, Inc., Denver, CO) for 1.5 h. Cells were then imaged using a ×63 objective on a Leica (Wetzlar, Germany) SP8 confocal microscope and several images were automatically stitched together using the Leica LASX software.

### Quantification of neuronal processes

The length of neuronal processes was measured using ImageJ as previously described^36^. Image scale was calibrated and the length of MAP2-positive processes was measured in four to five randomly selected fields (5–6 neurons per field) per treatment using ImageJ (NIH). Experiments were repeated three to four times using different preparations of primary cortical neurons. The number of neurons analyzed for each treatment ranged from 61 to 90. Investigators were blinded to treatment during analysis.

### Cell death

Cell death was measured using Hoechst/propidium iodide (PI) staining (cat#94403, cat#P4864, MilliporeSigma). After treatment in a 96-well plate, cell stains were added to the conditioned media at a concentration of 1 μg/ml (0.5 μl/well). The plate was then incubated at 37 °C for 5 min. Media were aspirated and cells were washed three to four times with 1× pre-warmed Hank’s balanced salt solution. Neurons were imaged on an Olympus IX-71 inverted epifluorescence microscope (Georgetown Medical Center Microscopy and Imaging Shared Resource) using the ×20 objective and cells were counted with ImageJ.

### Lactate dehydrogenase release assay

Lactate dehydrogenase (LDH) release was assayed using the CytoTox 96^®^ Non-Radioactive Cytotoxicity Assay (Promega, Madison, WI) according to the manufacturer’s protocols. Treatments were performed in a 96-well plate. At desired time point, 50 μl of media was collected from each well and transferred to a new 96-well plate. Non-conditioned media were used as a negative control. Fifty microliters of substrate solution was added to each well and incubated at RT for 30 min while protected from light. Then, 50 μl of stop solution was added to each well and the plate was read on a 96-well microplate reader (Bio-Rad, Hercules, CA) at 490 nm. All experiments were run in triplicate a minimum of three times.

### Western blot analysis

After treatment, conditioned media were aspirated and cells were gently rinsed with 1× PBS. Cells were collected in 1× RIPA (radioimmunoprecipitation assay) buffer (Merk Millipore Corp., Billerica, MA) with 1× Halt Protease/Phosphatase inhibitors (Thermo Fisher Scientific). Cells were maintained on ice for processing. After microtip sonication, lysates were centrifuged at ≥10,000 × *g* at 4 °C for 10 min. Protein concentration was determined by bicinchoninic acid (BCA) protein assay (cat#23225, Thermo Fisher Scientific). Lysates were loaded onto 4–12% Bis-tris gels (Thermo Fisher Scientific) for gel electrophoresis with ladders (cat#LC5800 or cat#LC5699, Thermo Fisher Scientific). After wet transfer (100 mV for 2 h) to 0.45 nm nitrocellulose membrane, the membrane was blocked for 30 min in 5% milk in PBS with 0.05% Tween-20 (PBST). Membranes were incubated with the following antibodies overnight at 4 °C: β-actin as a loading control (cat#A2228, 1:10,000, MilliporeSigma), HDAC6 (cat#7558, 1:2000, Cell Signaling), kinesin-1 heavy chain (cat#MAB1614, 1:1000, MilliporeSigma), and dynein intermediate chain (cat#MAB1618 1:1000, MilliporeSigma). Antibodies against acetylated tubulin (cat#T7451, 1:50,000, MilliporeSigma) and α-tubulin (cat#T5168, 1:50,000, MilliporeSigma) as an alternate loading control were incubated on membranes for 20 min at RT. After washing 3× for 5 min with PBST, incubation with corresponding HRP-conjugated anti-rabbit and anti-mouse secondary antibodies (cat# 111-035-003, 1:10,000, cat#115-035-003, 1:10,000, Jackson ImmunoResearch, West Grove, PA) occurred for 1 h at RT. Before reprobing, blots were stripped with Restore™ PLUS Western Blot Stripping Buffer (Thermo Fisher Scientific) for 10 min at RT and then examined for remaining chemiluminescence before re-blocking and probing with the next antibody.

### Transfection of primary neurons

Primary rat cortical neurons were plated at a density of 200,000 cells/ml. Cells were allowed to mature until DIV12. HDAC6 Silencer Select siRNA (small interfering RNA) (cat#4390771), scrambled siRNA (siSCR) (cat#4390843), and Lipofectamine RNAimax (cat #13378100) transfection reagent were purchased from Thermo Fisher Scientific. For each well of a 6-well plate (3 ml), neurons were treated with a mixture of final concentrations 10 nM for siRNA and 6 µl of RNAimax in filtered unmodified neurobasal media (NBM; cat#21103049, Thermo Fisher Scientific). BLOCK-iT™ AlexaFluor Red Fluorescent control (final concentration 20 nM; cat#14750100, Thermo Fisher Scientific) was used to evaluate transfection efficiency. The mixture was added to cells and incubated at 37 °C for 3 h. Media were removed and replaced with conditioned, pre-warmed NBM complete. It was allowed to sit overnight before beginning cell culture treatments.

### Co-immunoprecipitation

Cells were collected in RIPA buffer and protein content was immediately evaluated using BCA protein assay. An equal amount (100 µg) of each sample was loaded and brought to a final volume of 500 µl. Samples were precleared with 20 µl of Magnetic Protein A/G IgG (immunoglobulin G) beads (cat#88802, Thermo Fisher Scientific). Sample was removed from the beads and 5 µg of appropriate antibody was added: kinesin-1 heavy chain (cat#MAB1614, MilliporeSigma), dynein intermediate chain (cat#MAB1618, MilliporeSigma), or IgG control (cat#31903, Thermo Fisher Scientific). Samples and antibody were incubated overnight at 4 °C. Afterwards, the samples were added to 40 µl of new beads that had been blocked for 1 h at RT with 1% BSA in PBST. Beads and antibody-conjugated samples were placed on an end-over-end shaker at RT for 1 h. After incubation, flow through was removed and beads were rinsed three times with PBST. After a final wash with 1 ml UltraPure™ water (cat#10977015, Thermo Fisher Scientific), beads were incubated with 100 µl of 1× LDS Buffer (Thermo Fisher Scientific) for 10 min on the end-over-end shaker. After elution, sample was prepared for gel electrophoresis.

### Liquid chromatography-mass spectrometry

Liquid chromatography with tandem mass spectrometry (LC-MS/MS) was used to analyze co-immunoprecipitated (co-IP) tubulin. In brief, eluted co-IP samples in LDS Buffer were reduced (100 mM diothiothreitol (DTT), 3 h, 70 °C with shaking) and alkylated (125 mM iodoacetamide, 30 min in the dark at RT) before the reaction was stopped by 125 mM DTT. The samples were separated using gel electrophoresis. The 55 kDa bands were excised and cut into small pieces and destained, dried, and trypsinized for 16 h with 500 ng trypsin (Trypsin gold, MS grade, Promega) at 37 °C with agitation. Gel pieces were washed once (water/0.1% TFA), peptides were extracted three times (50% acetonitrile/0.1% TFA), and samples were dried in a Vacufuge plus (Eppendorf).

To relatively quantify tubulin in co-IP samples, samples underwent an on-bead trypsin digestion. Beads were washed twice (50 mM ammonium bicarbonate), reduced (5 mM DTT, 37 °C, 1 h), and alkylated (15 mM iodoacetomide, 30 min RT in the dark). Samples were then trypsinized with 500 ng trypsin (Trypsin gold, MS grade, Promega) at 37 °C with agitation. A second trypsinization step with 300 ng of trypsin for 4 h was carried out before peptides were removed from beads for MS analyses.

All LC-MS/MS analyses were carried out on a TripleTOF^©^ 6600 QTOF (quadrupole time of flight, Sciex) in positive ion mode with a NanoACQUITY UPLC (ultra performance liquid chromatography, Waters) attached. An analytical ACQUITY UPLC M-Class peptide BEH C18 column (300 Å, 1.7 µm, 75 µm × 15 cm, Waters) and a nanoACQUITY UPLC symmetry C18 trap column (100 Å, 5 µm, 180 µm × 20 mm, Waters) were used with mobile phases A (aqueous 2% acetonitrile, 0.1% formic acid) and B (acetonitrile, 0.1% formic acid) with a trapping flow rate of 15 µl/min and a analytical flow rate of 400 nl/min. All analytical runs were 60 min in length: 1 min at 99% solvent A and an increase of solvent B from 5 to 45% in 35 min increased to 99% solvent B in 2 min and held for 3 min before returning to 99% solvent A for 20 min. Declustering potential was set to 80 and ionspray voltage 2300.

The LC-MS/MS method for protein identification of in-gel bands used a top 30 data-dependent acquisition method. TOF MS accumulation time was 250 ms for 400–2000 Da. The TOF MSMS accumulation time was 50 ms for 100–1800 Da; the intensity threshold of 100 based on the background and exclusion after two MS/MS of 5 s was based on the peak width. Peak list generating and protein identification searches were carried out by Protein Pilot Software™ 5.0.1.0. The Uniprot *Rattus norvegicus* database, with the addition of IgG *Mus musculus*, including the contained 8064 sequences and the same number of reversed sequences, was used and settings were as per the 6600 QTOF.

Multiple reaction monitoring-high resolution (MRM-HR) method was used for protein quantification; this approach targeted and optimized for α-tubulin and β-tubulin peptides. Collision energy (CE) were optimized for six peptides: four from α-tubulin (DVNAAIATIK, *m/z* 508.2918, CE 30; TIGGGDDSFNTFFSETGAGK, *m/z* 669.9692, CE 25; NLDIERPTYTNLNR, *m/z* 573.6322, CE 30; EIIDLVLDR, *m/z* 543.3137, CE 25) and two from β-tubulin (GHYTEGAELVDSVLDVVR, *m/z* 653.6655, CE 25; EVDEQMLNVQNK, *m/z* 723.8483, CE 35). A CE spread of 15 was used for all peptides and a dwell time of 100 ms for each peptide was used giving a cycle time that allowed at least 10 points on the curve for each peak. A 35 min blank was run after every sample to stop any unexpected carry over interference. A trypsinized β-galactosidase standard was run following every third sample. Sample order was randomized. MultiQuant™ software version 2.1.1 (Sciex, Framingham, MA) was used for quantitation. Quantitation was based on fragment mass and the addition of the top four fragments. Mass allowance was ±0.05 Da. IgG-negative control was subtracted from other samples.

### Microfluidic chamber and coverslip preparation and assembly

Coverslips (cat#48393060, VWR, Radnor, PA) were washed overnight with 70% nitric acid (MilliporeSigma) and then washed with water and flamed to sterilize. Each coverslip was coated with poly-d-lysine (0.05 mg/ml, cat#P9155, MilliporeSigma) overnight at 37 °C. Coverslips were then rinsed three times in MilliQ water and dried completely. Coverslips were placed in a 60 mm Petri dish for assembly.

Microfluidic chambers (cat#SND450, Xona Microfluidics, Temecula, CA) were cleaned with a 1% Alconox solution (w/v%) in MilliQ water, rinsed six times with MilliQ water, dipped in 70% ethanol, and left to dry in cell culture hood for 20 min under ultraviolet light. Sterile chambers were then placed on glass coverslips pre-coated with poly-d-lysine. Primary rat cortical neurons were prepared as described above. Cells were plated at a density of ~50,000 neurons per chamber in a plating media (Neurobasal complete + 10% FBS) and a higher volume of media was maintained in the axonal compartment. After 12–18 h, a full media exchange was completed and media were replaced with NBM complete. Neurons normally extend axons through the microgrooves into the axonal compartment at DIV4/5. From this time until use (DIV7), higher volume of media was maintained in the soma compartment.

### Live imaging-quantum dot BDNF

Using commercially available microfluidic chambers (Xona Microfluidics), primary rat neurons were grown until DIV7 as previously described above. At this maturity, the BDNF cargo moves in both the retrograde and anterograde direction, with a slight preference for retrograde movement. The microgroove width of 450 μm allows axons to extend to the adjacent compartment, but excludes dendrites^37^. Monobiotinylated, biologically active, BDNF was prepared as previously described^38^ and was incubated with Qdot™ 655 Streptavidin Conjugate at a molar ratio of 1:1 (cat#Q10123MP, Thermo Fisher Scientific). Quantum dot-labeled monobiotinylated BDNF (Qdot-BDNF) was added to the axonal compartment of microfluidic chambers to a final concentration of 1 nM.

After establishing baseline movement of Qdot-BDNF, 5 nM of gp120 or boiled (heat-inactivated) gp120 were added to the microfluidic chamber for 2 h and imaged with a ×63 oil objective using a Leica SP8 microscope in an environmental chamber at 37 °C/5%CO_2_. Time-lapsed image series were captured at a frame rate of 500 ms/frame for 2 min at 15 min intervals. Microgrooves without axons served as a negative control for background staining.

### Analysis of Qdot movement

Images were processed in ImageJ and Qdot-BDNF were traced using the Manual Tracking Plugin. Data were then exported and analyzed for velocity, directionality, pausing, and separated based on directionality. Quantum dots were excluded from analysis if paused more than 60% of the time to ensure only those BDNF molecules attached to motor proteins were analyzed.

### Statistical analysis

Statistical analyses were performed using the GraphPad Prism 8 Software (GraphPad Software, Inc., La Jolla, CA). Results are depicted as mean ± standard error of mean (SEM). For a comparison of more than two groups, a one-way analysis of variance (ANOVA) test, followed by a proper post hoc test for multiple comparisons, was applied as indicated in the figure legends. Sample size ranging from three to six independent preparations of primary neurons was chosen based on previous experience to detect the hypothesized effect size. Data were analyzed and meet assumptions of normality and equal variance with the Shapiro–Wilk normality test and the Brown–Forsythe test, respectively.

Eight animals per group were utilized based on power analysis to detect an effect size of 30% as obtained from in vitro preliminary data. A two-tailed, paired *t* test was used to compare to vehicle-injected contralateral controls. *P* values <0.05 indicate statistical significance.

## Results

### HIV and gp120 cause deacetylation of neuronal tubulin

**Fig. 1:**
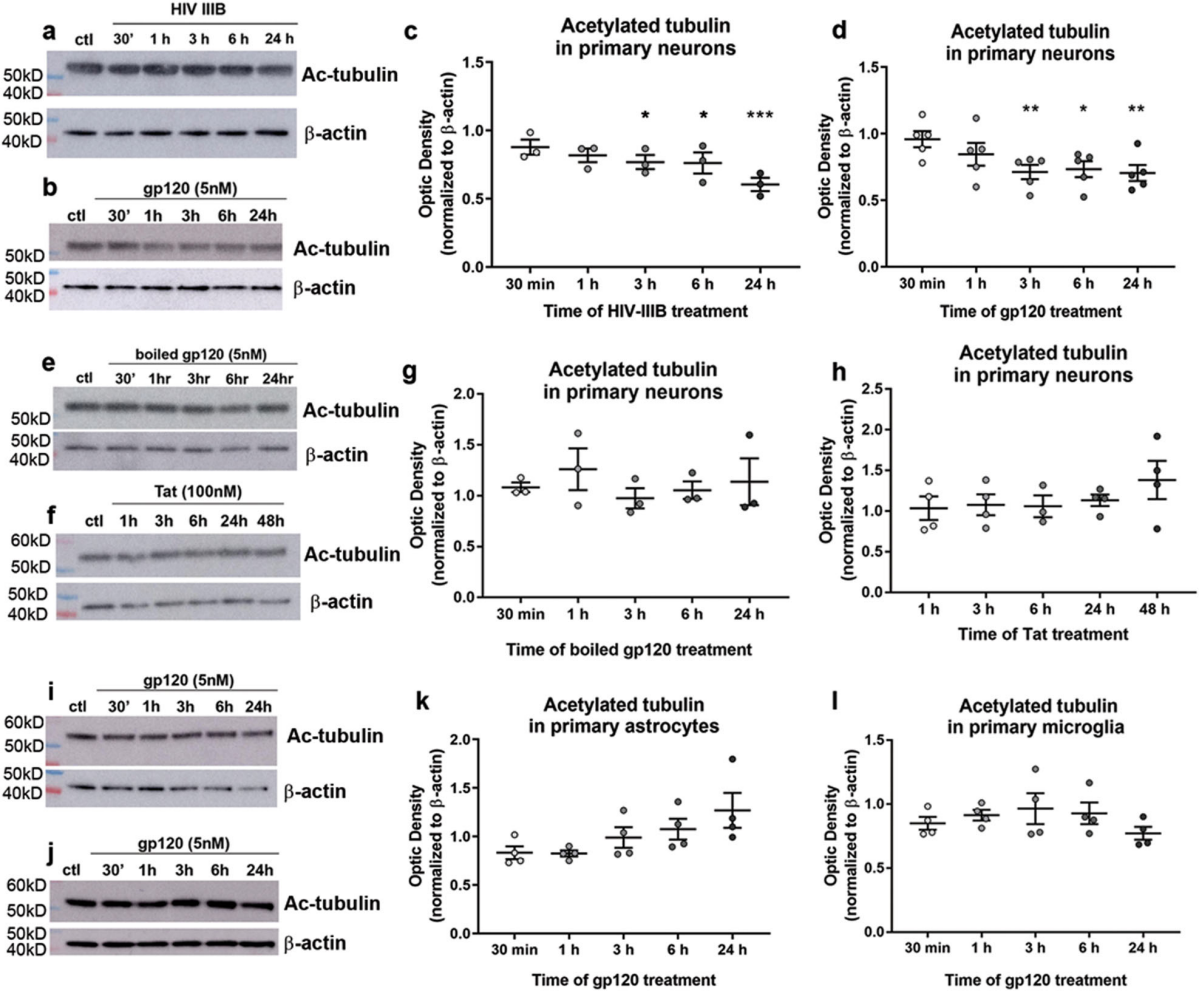
HIV and gp120 cause deacetylation of tubulin in primary rat neurons. Primary rat cortical neurons were exposed to either HIVIIIB (**a**, **c**), gp120 (**b**, **d**), boiled gp120 (**e**, **g**), or Tat (**i**, **k**) for the indicated time points. Primary rat astrocytes (**i**, **k**) or microglia (**j**, **l**) were exposed to gp120 for 30 min up to 24 h. Cell lysates were then collected and examined by Western blot for acetylated tubulin (Ac-tubulin) immunoreactivity. β-Actin was used as a loading control. Dots indicate independent experiments. Bars indicate mean ± SEM, *p < 0.05, **p < 0.01, and ***p < 0.001 vs. control; one-way ANOVA with Dunnett’s multiple comparisons post hoc

HIV transgenic rats, which express seven of nine HIV proteins including gp120^39^, have decreased acetylated tubulin in the cortex at both 5 and 9 months of age when compared to wild-type controls^27^. Acetylation of tubulin often alters the functionality of microtubules. However, little is known about whether the decrease in acetylated tubulin is neuron specific and what role viral proteins play in this effect. To examine whether the deacetylation of tubulin selectively occurs in neurons and whether its effect is due to the HIV envelope protein gp120, we exposed primary rat cortical neurons to HIVIIIB (3 ng/ml of p24), gp120IIIB (5 nM), or boiled/heat-inactivated gp120 as a negative control (5 nM) for 30 min up to 24 h and measured levels of acetylated tubulin by Western blot analysis. We found that HIVIIIB elicited a time-dependent decrease in acetylated tubulin beginning by 3 h (Fig. 1a, c). Exposure of neurons to gp120 (Fig. 1b, d), but not to heat-inactivated gp120 (Fig. 1e, g), also decreased acetylated tubulin beginning at 3 h and up to 24 h. Further, we exposed neurons to another neurotoxic viral protein Tat^40^ for up to 48 h to examine whether other viral proteins affect tubulin acetylation. Tat failed to change the levels of acetylated tubulin (Fig. 1f, h). Therefore, it appears that gp120 likely plays a crucial role in HIV-mediated deacetylation of tubulin.

Next, we aimed to determine whether gp120’s effect occurs exclusively in neurons, since tubulin is a key protein in the cytoskeleton of all cells within the central nervous system. Exposure of primary rat astrocytes (Fig. 1i, k) or microglia (Fig. 1j, l) to gp120 for up to 24 h failed to change the levels of acetylated tubulin. These data suggest that the effect of HIV- and gp120-mediated deacetylation is specific to neuronal tubulin.

### Gp120 increases the levels of HDAC6 in vitro and in vivo

**Fig. 2:**
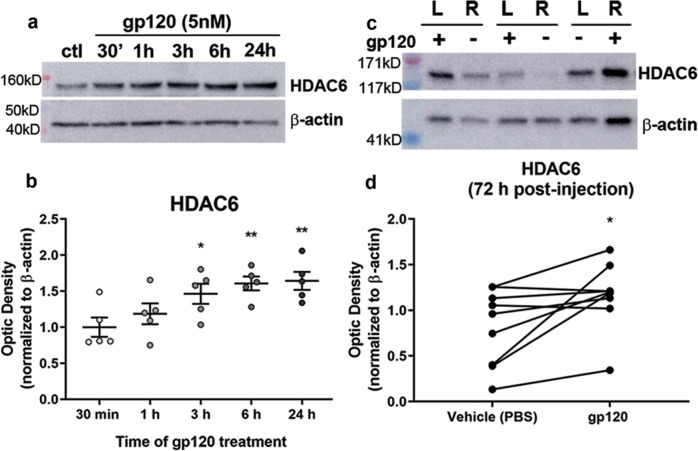
Gp120 causes increased HDAC6 in vitro and in vivo. **a**, **b** Primary rat cortical neurons were exposed to gp120 (5 nM) for the indicated time points. Cell lysates were collected and examined for HDAC6 immunoreactivity by Western blot (**a**). β-Actin was used as a loading control. **b** Optical density of HDAC6 immunoreactivity normalized by β-actin. Bars indicate mean ± s.e.m (*n* = 5). *p < 0.05, **p < 0.01 vs. control, one-way ANOVA with Dunnett’s multiple comparisons post hoc. **c**, **d** Rats were injected into the striatum with gp120 (400 ng) and contralaterally with vehicle, and were euthanized 72 h post injection. **c** Example of Western blot analyzed for HDAC6. “L” indicates left hemisphere, “R” indicates right hemisphere, “+” indicates gp120 injection, and “−” indicates vehicle control injection. **d** Quantification of optical density. Lines connect the values from ipsilateral and contralateral striatum within the same animal. Some dots in the vehicle group overlap because the values were similar. *P < 0.05; two-tailed, paired *t* test. Rats injected with gp120 and euthanized 24 h later did not show an increase in HDAC6 (not shown)

Further examination into the mechanism of gp120-mediated deacetylation of neuronal tubulin is essential in order to discover new therapeutic interventions. Deacetylation of tubulin is mainly mediated by HDAC6^16,19^. An increase in expression or activity of this enzyme would decrease the levels of acetylated tubulin. Thus, we tested whether gp120 changes the expression of HDAC6 by exposing primary rat cortical neurons to gp120 for up to 24 h and assessing HDAC6 levels by Western blot analysis. We observed a time-dependent increase in HDAC6 immunoreactivity starting by 3 h (Fig. 2a, b). This time point coincides with gp120-mediated decrease in acetylated tubulin, suggesting that HDAC6 could likely be the enzyme mediating gp120-induced deacetylation of tubulin.

To determine whether the gp120-mediated increase in HDAC6 is reproducible in vivo, rats received an injection of vehicle control either in the right or left striatum and 400 ng of gp120IIIB into the contralateral striatum. This model of striatal delivery of gp120 has been previously utilized to produce a time-dependent increase in activated caspase-3 expression in neurons, starting by 4 days after injection^41^. To evaluate whether gp120 increased HDAC6 before significant cell death, animals were euthanized 72 h after the injections, the striata were dissected, and HDAC6 levels were evaluated using Western blot. Striatal levels of HDAC6 were significantly increased by gp120 (Fig. 2c, d). Thus, we could conclude that this enzyme is likely responsible for gp120-mediated decrease of acetylated tubulin.

### Inhibition of HDAC6 reverses gp120-mediated decrease in acetylated tubulin

**Fig. 3:**
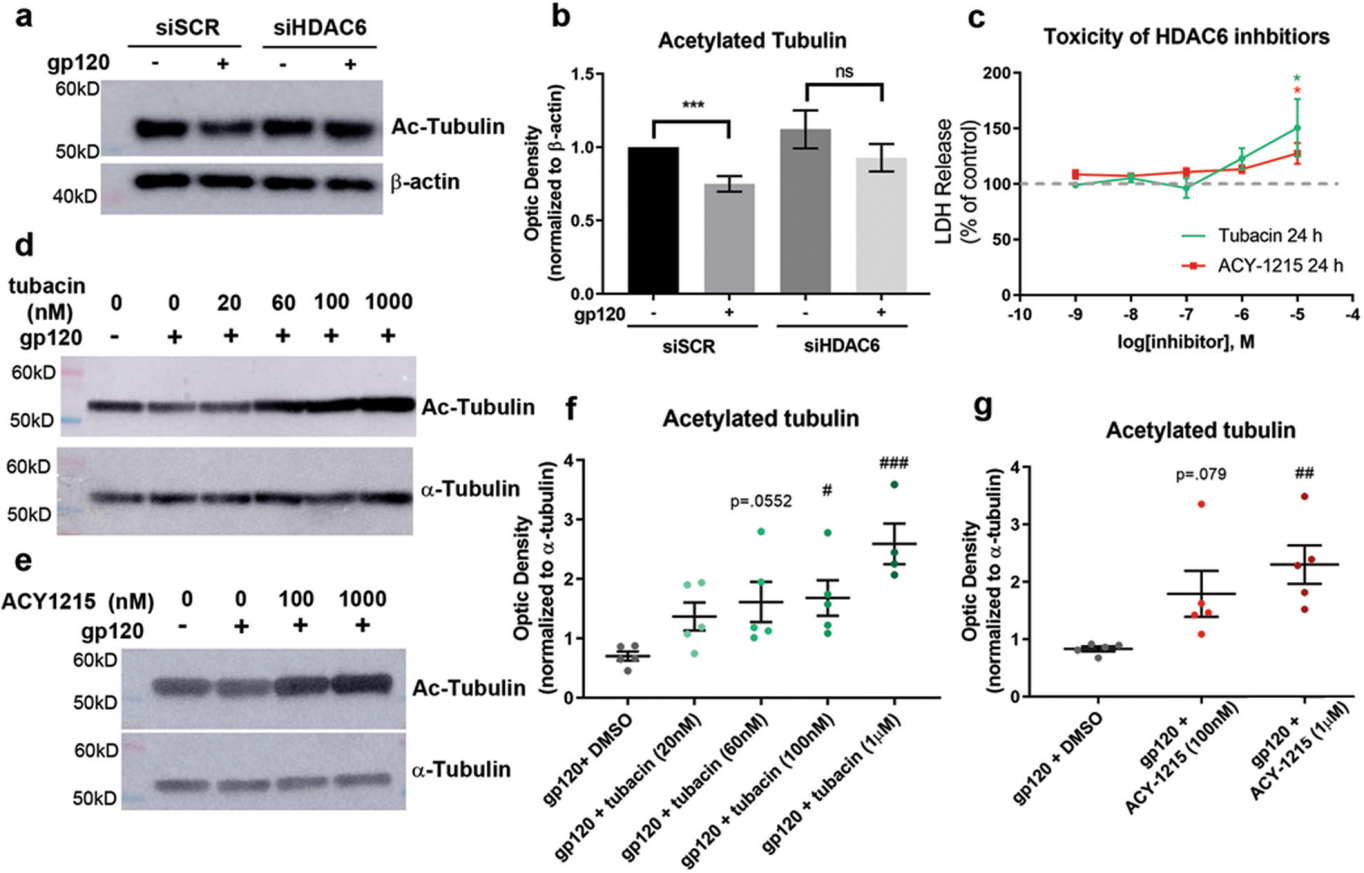
Inhibition of HDAC6 prevents gp120-mediated deacetylation of tubulin. **a**, **b** Neurons were transfected with siHDAC or siSCR. Two days later, neurons were exposed to boiled gp120 or gp120 (5 nM) for 24 h. Lysates were then analyzed for Ac-tubulin by Western blot. ***p < 0.001 vs. untreated control transfected with the same vector; two-sided, Student’s *t* test. **c** Lactate dehydrogenase (LDH) release was measured from neurons treated with indicated doses tubacin and ACY-1215. ***P < 0.001 vs. control, one-way ANOVA with Dunnett’s multiple comparisons post hoc. **d**, **f** Primary rat cortical neurons were exposed to gp120 alone or in combination with sub-toxic doses of tubacin and **e**, **g** ACY-1215 for 24 h. Cell lysates were collected and analyzed by Western blot for Ac-tubulin immunoreactivity. α-Tubulin was used as a loading control. ^#^*P* < 0.05, ^##^*p* < 0.01, ^###^*p* < 0.001 vs. gp120, one-way ANOVA with Tukey’s post hoc. Bars indicate mean ± SEM. Data points represent independent cultures of primary neuron. ns not significant

To establish that HDAC6 is the key enzyme responsible for the gp120-mediated deacetylation of tubulin, we utilized an siRNA approach to decrease levels of HDAC6 in neurons. Primary rat cortical neurons were transfected with HDAC6 siRNA (siHDAC6) or siSCR as a control. Two days later, cell lysates were collected, and HDAC6 levels were evaluated via Western blot. Neurons transfected with siHDAC6 expressed an average of 42% less HDAC6 than those transfected with siSCR (Supplementary Fig. 1). Neurons transfected with siHDAC and siSCR were exposed to gp120 for 24 h and acetylated tubulin levels were examined. We found that partial silencing of HDAC6 expression abolishes the significant decrease in acetylated tubulin seen in gp120-treated neurons (Fig. 3a, b).

To further support the relationship between HDAC6 inhibition, acetylated tubulin, and neuronal survival, neurons were exposed to two potent and selective inhibitors of HDAC6 activity, tubacin (half-maximal inhibitory concentration (IC_50_) = 4 nM) and Ricolinostat (ACY-1215; IC_50_ = 5 nM). First, we evaluated the toxicity of tubacin and ACY-1215 to primary rat cortical neurons by measuring LDH release, a marker for cell damage. Neurons were exposed for 24 h to several concentrations of tubacin or ACY-1215 ranging from 1 nM to 10 μM. There was no significant increase in LDH release 24 h after tubacin or ACY-1215 application at concentrations at or <1 μM, whereas treatment with 10 µM of either inhibitor resulted in a significant increase of LDH release (Fig. 3c). Therefore, for the continuation of this study, we utilized non-toxic concentrations of HDAC6 inhibitors (between 20 nM and 1 µM) to examine the neuroprotective effect of HDAC6 inhibition. Primary rat cortical neurons were exposed to gp120 alone or in combination with tubacin or ACY-1215 for 24 h. Both tubacin (Fig. 3d, f) and ACY-1215 (Fig. 3e, g) prevented the gp120-mediated decrease in acetylated tubulin in a concentration-dependent manner.

### HDAC6 inhibition protects against gp120-induced neurotoxicity

**Fig. 4:**
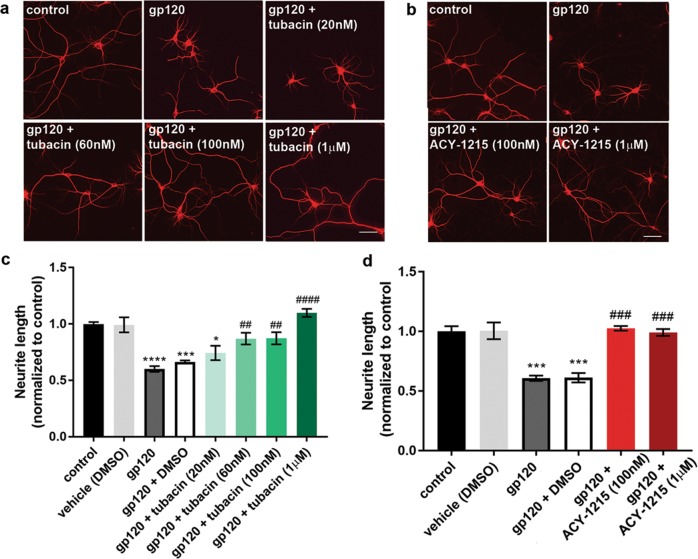
HDAC6 inhibition prevents gp120-mediated neurite shortening. **a** Primary rat cortical neurons (DIV14) were exposed to boiled gp120 (control), gp120 alone (5 nM), or in combination with tubacin or **b** ACY-1215 for 24 h. Cells were fixed, stained for MAP2, and imaged at ×20. Scale bar: 50 nm. **c**, **d** Neurite processes were quantified using ImageJ. Data are presented as mean ± SEM, *p < 0.05, ***p < 0.001, ****p < 0.0001 vs. control, ^##^*p* < 0.01, ^###^*p* < 0.001, ^####^*p* < 0.0001 vs. gp12^0^ one-way ANOVA with Tukey’s post hoc. Experiments were repeated with four independent cultures of neurons for each drug treatment

Previous studies have reported that deacetylation of tubulin causes shortened MAP2-positive neurite processes^42^, a phenomenon that has also been described to occur when neurons are exposed to gp120^13^, or in postmortem frontal cortex of HAND subjects^43^. Shortened and simplified processes are indicators of declining neuronal health and are a pre-apoptotic marker of cellular stress^44^. Therefore, to establish whether inhibition of HDAC6 is protective against gp120-mediated neurotoxicity, neurons were exposed to gp120 alone or in combination with tubacin (20 nM–1 µM) or ACY-1215 (100 nM or 1 µM) for 24 h and stained using MAP2 to identify neurites (Fig. 4a, b). We observed that concentrations of tubacin at 60 nM or higher (Fig. 4c) and ACY-1215 (Fig. 4d) at both 100 nM and 1 μM prevent gp120-mediated shortening of neurites.

**Fig. 5:**
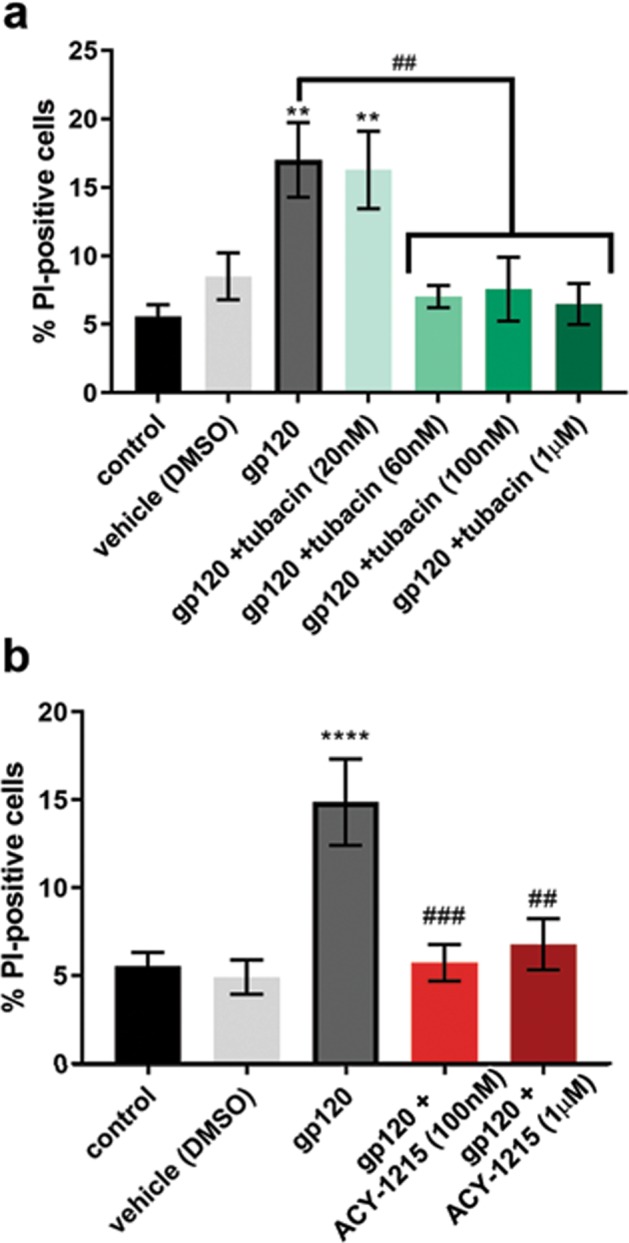
HDAC6 inhibition prevents gp120-mediated cell death. Primary rat cortical neurons (DIV14) were exposed to gp120 alone (5 nM) or in combination with **a** tubacin or **b** ACY-1215 for 24 h. Cell loss was determined by Hoechst/PI staining. Data are presented as mean ± SEM, **p < 0.01, ****p < 0.0001 vs. control, ^##^*p* < 0.01, ^###^*p* < 0.001 vs. gp120; one-way ANOVA with Tukey’s post hoc. Experiments were repeated with five independent cultures of neurons for each drug treatment

To determine if pharmacological inhibition of HDAC6 rescues gp120-mediated neuronal cell death, primary rat cortical neurons were exposed to gp120 alone or in combination with various concentrations of tubacin or ACY-1215 for 24 h. Neurons were stained for Hoechst/PI to examine cell survival. As expected, gp120 caused an increase in PI-labeled cells when compared to control, denoting increased neuronal loss. This effect was prevented by both tubacin (Fig. 5a) and ACY-1215 (Fig. 5b) in a concentration-dependent manner, indicating that pharmacological inhibition of HDAC6 is neuroprotective against gp120-mediated neurotoxicity.

### Gp120 decreases motor protein–tubulin interactions

**Fig. 6:**
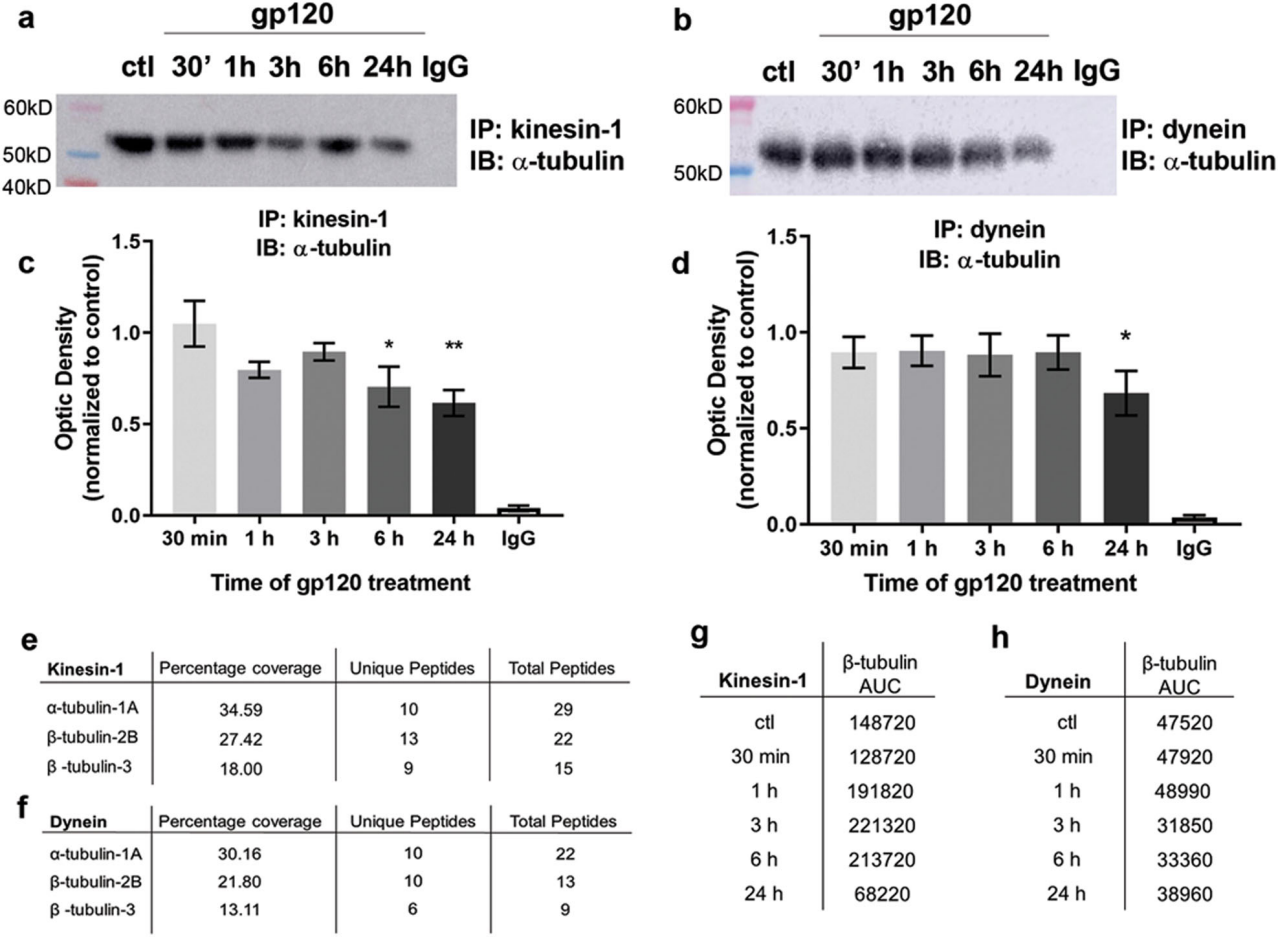
Gp120 causes decrease in motor protein and tubulin interactions. Primary rat cortical neurons (DIV14) were exposed to boiled gp120 (ctl) or gp120 (5 nM) for the indicated time points. Cell lysates were prepared and co-immunoprecipitated using kinesin (**a**) or dynein (**b**) antibodies. Immunoprecipitated proteins were analyzed by Western blot using α-tubulin antibody. Data are presented as mean ± SEM, *p < 0.05, **p < 0.01 vs. control; one-way ANOVA with Dunnett’s multiple comparisons post hoc. Experiments were repeated with five independent cultures of neurons for each drug treatment. **e**, **f** LC-MS/MS identification of tubulin in co-IP samples. Tubulin was identified in the 55 kDa band of the **e** kinesin-1 and **f** dynein co-IP; data ares from the 0 min time point. Only peptides identified at the 95% confidence level are included in the percentage coverage (percentage of protein amino acid sequence that was identified), unique peptides (number of unique peptides identified), and total peptides (total number of peptides identified including replicates). **g**, **h** Relative quantitation by MRM-HR using the β-tubulin peptide GHYTEGAELVDSVLDVVR. β-Tubulin quantification is shown as area under the curve (AUC)

Previous studies have shown that deacetylation of tubulin causes decreased association between tubulin and the motor proteins kinesin-1^22^ and dynein^21^. The diminished association between these proteins may lead to impaired axonal transport. Thus, we hypothesized that gp120-mediated neurotoxicity encompasses a diminishing association of motor proteins with tubulin. To test this hypothesis, we exposed primary neurons to gp120 for various time points and performed co-IP using antibodies against kinesin-1 (Fig. 6a, c) or dynein (Fig. 6b, d) and probed with α-tubulin. Compared to control cells, in gp120-treated cells there was a time-dependent decrease in tubulin immunoreactivity (55 kDa band) when both kinesin and dynein antibody were used for co-IP. The decrease is not due to alterations in motor protein availability as there were no differences in either kinesin-1 or dynein immunoreactivity following gp120 treatment (Supplementary Fig. 2). Thus, our data suggest that gp120 decreases the association between kinesin-1/dynein and tubulin.

To confirm that tubulin is co-immunoprecipitated with motor proteins, the 55 kDa band was analyzed by LC-MS/MS. At least three tubulin isoforms were identified in both the kinesin-1 (Fig. 6e) and dynein (Fig. 6f) co-IPs, α-tubulin, and β-tubulins 2B and 3. Moreover, as expected, given that a large quantity (5 µg) of antibody was used for each immunoprecipitation, we also identified mouse IgG-2B within the samples (data not shown).

To support the decreased association of motor protein and tubulin in the co-immunoprecipitated samples, a targeted MS method was utilized with a conserved peptide for β-tubulin. Over the 24 h time course of gp120 treatment, the kinesin-1 co-IP showed an initial decrease of β-tubulin by 30 min when compared to control, followed by an increase between 1 and 6 h (Fig. 6g). By 24 h, gp120 caused an ~50% decrease in co-IP β-tubulin (Fig. 6g), similar to the results shown by Western blot for α-tubulin. For the dynein co-IP, a decrease in β-tubulin by gp120 was observed by 3 h (Fig. 6h).

### Gp120 impairs axonal transport of BDNF

**Fig. 7:**
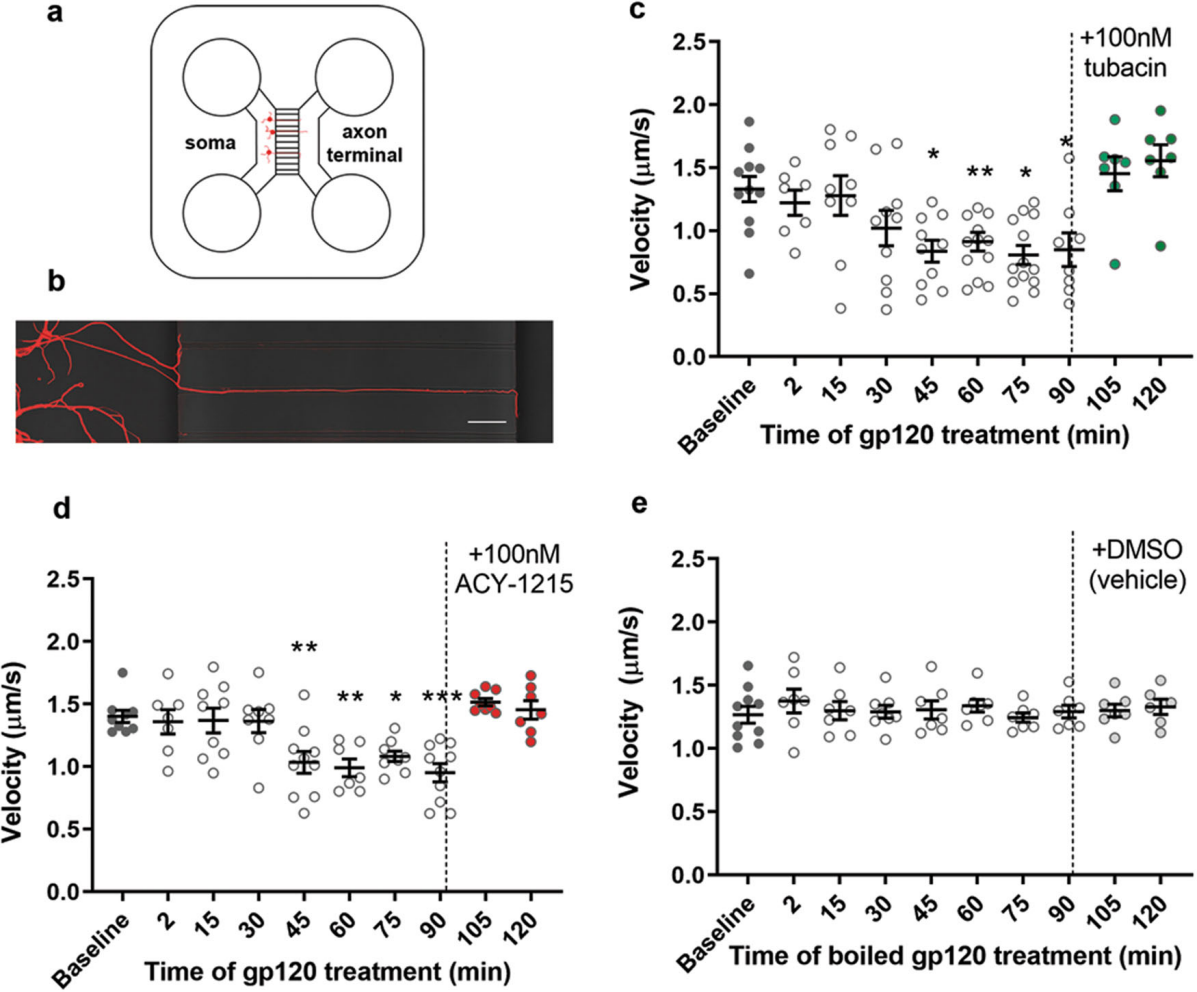
Gp120 causes a decrease in axonal transport of BDNF, which is rescued with HDAC6 inhibition. **a** Schematic of microfluidic chamber where cells are plated on one side (somal) and extend their axons into the axonal side. The length of the microfluidic chamber is 450 µm. **b** Enlargement of neuron growing through microfluidic chamber, stained with tubulin, and imaged at ×63. Scale bar: 50 µm. **c**, **d** After imaging for baseline velocity, chambers were exposed to gp120 (5 nM) for 2 h. After 90 min, **c** tubacin (100 nM) or **d** ACY-1215 (100 nM) was added as indicated by the dotted black line. **e** Chambers were treated with boiled/heat-inactivated gp120 for 2 h. After 90 min, vehicle control (DMSO) was added as indicated by the dotted black line. Bars indicate mean ± SEM. Individual data points represent one tracked Qdot-BDNF molecule. *P < 0.05, **p < 0.01, ***p < 0.001 vs. baseline control; one-way ANOVA with Dunnett’s multiple comparisons post hoc. Experiments were repeated with five and four independent cultures of neurons for tubacin and ACY-1215, respectively. Control experiments were done in three independent cultures

The relationship between acetylation status of microtubules, inefficiency of motor protein binding to tubulin, and impaired axonal transport has been seen in experimental models of other neurodegenerative diseases, including Alzheimer’s^45^ and Huntington’s diseases^21^. To evaluate the functional outcomes of gp120-mediated tubulin deacetylation and HDAC6 inhibition, we measured axonal transport in live cells by using a Qdot-BDNF. BDNF is a neurotrophic factor that is both anterogradely and retrogradely transported by the motor proteins kinesin-1 and dynein, respectively^24,46^. Qdot-BDNF allows for sensitive visualization of axonal transport via live imaging of neurons grown on microfluidic chambers^38^, which identify and separate axons from dendrites^37^. Figure 7a, b show a schematic and an example of a tubulin-stained neuron grown in our microfluidic chambers.

Baseline recordings for Qdot-BDNF velocity were made for 15 min before the treatment of gp120. The average velocity of BDNF cargo at baseline was 1.33 µm/s, similar to baseline speed previously reported for this construct^38^, suggesting fast-axonal transport. Neurons were then exposed for 90 min to gp120 or boiled gp120 (as a control), and 2 min of live imaging were taken every 15 min. Analysis was completed using the Manual Tracking plugin for ImageJ (see Materials and methods). Gp120 causes a time-dependent decreased velocity of Qdot-BDNF starting by 45 min and up to 90 min (Fig. 7c, d). Both anterograde and retrograde velocity was equally impaired by gp120 (Supplementary Fig. 3). After 90 min of gp120 exposure, either 100 nM of tubacin (Fig. 7c) or ACY-1215 (Fig. 7d) was added to the chambers. The same axon was then imaged for 30 additional minutes using the same parameters as above. Both tubacin (Fig. 7c) and ACY-1215 (Fig. 7d) restored Qdot-BDNF velocity to baseline levels 15 min after their addiction to neuronal cultures. The effect of these HDAC6 inhibitors was maintained for up to 30 min, despite the continued presence of gp120 (Fig. 7c, d). No changes in velocity were observed when neurons were exposed to boiled gp120 or dimethyl sulfoxide (Fig. 7e). Overall, our data suggest that the deficits in axonal transport caused by gp120 can be reversed by HDAC6 inhibitors.

## Discussion

The results obtained from this study suggest a novel mechanism of HIV neurotoxicity that relies on gp120’s ability to impair axonal transport by decreasing the acetylation of neuronal tubulin. Importantly, the neurotoxic effect of gp120 is reversed by both tubacin and ACY-1215, suggesting that HDAC6 inhibition may be a viable therapeutic strategy to reduce synaptopathy and neuronal damage seen in HAND subjects.

HIV does not infect neurons; nevertheless, it produces neuronal damage and synaptic loss when added to neurons in culture. We have previously shown that the neurotoxic effect of HIV is blocked by CCR5 or CXCR4 receptor antagonists^47^, supporting the notion that HIV sheds gp120, which then acts independently from the virus and promotes neurotoxicity by binding to these chemokine receptors^9^. The envelope protein is endocytosed into neurons^11–13^. Once internalized, gp120 binds to the C-terminal tail domain (CTT) of class III β tubulin (TUBB3)^14^, a neuronal-specific tubulin. Thus, one may speculate that gp120’s ability to decrease tubulin acetylation is a consequence of the binding to TUBB3. Indeed, Helix-A, a peptide that displaces gp120 from binding to TUBB3, prevents both gp120-mediated decrease in tubulin acetylation and the neurotoxic effect of gp120, irrespective of whether M- or T-tropic strain is used^14^. This is also consistent with previously reported data indicating similar relative potencies of M- and T-tropic strains of gp120 in promoting neurodegeneration^14,27^. Further, gp120, by binding to the CTT of TUBB3, could provide steric hindrance to residues important for strong binding of motor proteins and tubulin^48^. When the CTT of tubulin is cleaved, motor processivity or run length of both kinesin and dynein is decreased^49,50^. Additional interference might be seen with MAPs such as MAP2 and tau, which also bind on the CTT of tubulin^51^. In this study, we demonstrate that gp120 decreases the association of kinesin-1 and dynein to microtubules. These motor proteins are critical for the trafficking of organelles and vesicles in neurons. Thus, impaired binding of kinesin-1 and dynein to microtubules could compromise axonal transport, which is essential for neuronal function and survival^52^.

Binding of gp120 to tubulin and the subsequent alteration of motor protein association with microtubules are appealing mechanisms to explain gp120’s toxicity. However, we cannot exclude the possibility that CXCR4 or CCR5 receptor-mediated signaling may be altering motor protein function. For instance, glycogen synthase kinase-3β (GSK3β), which is downstream of CXCR4 activation^53^, phosphorylates kinesin light chains and inhibits anterograde transport^54^. However, GSK3β signaling does not impair retrograde transport, which is an effect seen after exposure to gp120 (Supplementary Fig. 3). In fact, gp120 causes a decrease in the velocities of both anterograde and retrograde transport, measured by Qdot-BDNF trafficking. Therefore, we suggest that GSK3β signaling is not the initial mechanism that triggers the observed impairment in bidirectional axonal transport of BDNF.

One additional finding reported here is the lack of effect of another viral protein Tat on tubulin deacetylation. This is surprising because, similar to gp120, Tat is endocytosed and axonally transported, and promotes neurotoxicity^55^. Despite not altering acetylation of tubulin, Tat may impair neuronal microtubules using a different mechanism such as increasing proteasome-mediated degradation of MAP2, and therefore decreasing support for microtubules^56^. Tat also has several mechanisms of neurotoxicity that do not rely on modification of the neuronal cytoskeleton including modifications to intracellular Ca^2+^ by phosphorylation of glutamate receptors^57^. Moreover, Tat impairs mitochondrial dynamics, but not necessarily transport, in neurons, which also may explain its neurotoxic effect^58^.

The role that HDAC6 plays in gp120 neurotoxicity was tested by both siRNA and HDAC6 inhibitors. These inhibitors could also have off-target effects and be neuroprotective by mechanisms that involve more than just microtubule acetylation. HDAC6 acts primarily in the cytoplasm and has a multitude of targets^16^. These targets regulate several processes within cells, including inflammation, protein degradation, and transcription, as well as cell signaling and cytoskeletal components^59^. Therefore, HDAC6 inhibition could promote acetylation of many targets in order to elicit its neuroprotective effect. For example, HDAC6 deacetylates nine lysine residues on cortactin, a protein that can promote rearrangement and polymerization of the actin cytoskeleton. Maintaining the acetylation of cortactin, such as through HDAC6 inhibition, improves postsynaptic density protein 95 clustering^60^, which is crucial for the formation of functional dendritic spines and regulation of excitatory synapses. These synapses are often impaired, either in structure or number, in HAND subjects^43,61^ as well as other neurodegenerative diseases^62^ even without overt neuronal injury. Gp120 causes a decrease in the number of dendritic spines^63^ and thus HDAC6 inhibition may counteract not only the toxic effect of gp120 on microtubules but also improve the number of functional dendritic spines.

Overexpression of HDAC6 induces pro-inflammatory responses through nuclear factor-κB signaling in macrophages^64^. Conversely, inhibition of HDAC6 prevents the activation of macrophages and the release of pro-inflammatory cytokines after stimulation by lipopolysaccharide^65^ or the HIV protein Tat^66^. These considerations, taken together with our data, suggest that HDAC6 inhibition may be a viable therapeutic strategy to reduce inflammation and neuronal damage seen in HAND subjects. Indeed, some HIV-positive patients still exhibit inflammation and activated macrophages/microglia despite ART and viral suppression^67,68^. Thus, we hypothesize that the inhibition of HDAC6 in patients with pro-inflammatory responses due to HIV or HIV proteins may also be beneficial. Further examination into the effect of HDAC6 inhibition in vivo should be completed to determine whether this mechanism is a viable therapeutic target to prevent the neuronal damage seen in HAND. In vitro, HDAC6 also regulates several aspects of HIV infection. HDAC6 promotes the deacetylation of Tat and suppresses its transactivation activity^69^, as well as regulates the fusion activity of gp41/gp120 protein^70^. Therefore, HDAC6 inhibition in vivo might increase the efficiency of HIV replication (reviewed in ref. ^71^). In fact, ongoing clinical trials are examining whether HDAC inhibitors will decrease viral latency in the periphery.

In this study, we sought to provide evidence about the pathological significance of gp120 binding to tubulin CTT and decreased tubulin acetylation in neurons. Acetylation is a post-translational modification of tubulin that occurs frequently in the proximal portion of axons within mature neurons. Increased tubulin acetylation promotes the activity of motor proteins and, consequently, improves axonal transport^72,73^. In this work, we demonstrated that one consequence of decreased acetylated tubulin caused by gp120 is impairment in the fast-axonal transport of BDNF. When levels of acetylated tubulin are restored through HDAC6 inhibition, we observed the return of axonal transport of BDNF to baseline velocity even in the continued presence of gp120. Thus, HDAC6 inhibition is sufficient to rescue neurons from the neurodegenerative effects of gp120. Although our data were obtained with an experimental model of neuronal damage, providing new observations about the potential causes of HAND will support the development of adjunct neurological therapies. This is an important issue because although ART reduces viral load and neurodegeneration^43^, it does not abolish HAND^74,75^.

## Supplementary information

Supplementary Figure 1

Supplementary Figure 2

Supplementary Figure 3
