# Supplementary figures and images for: Histone deacetylase 6 inhibition rescues axonal transport impairments and prevents the neurotoxicity of HIV-1 envelope protein gp120
Source: Cell Death Dis. 2019 Sep 12;10(9):674. doi: 10.1038/s41419-019-1920-7 (PMC6742654; doi:10.1038/s41419-019-1920-7)

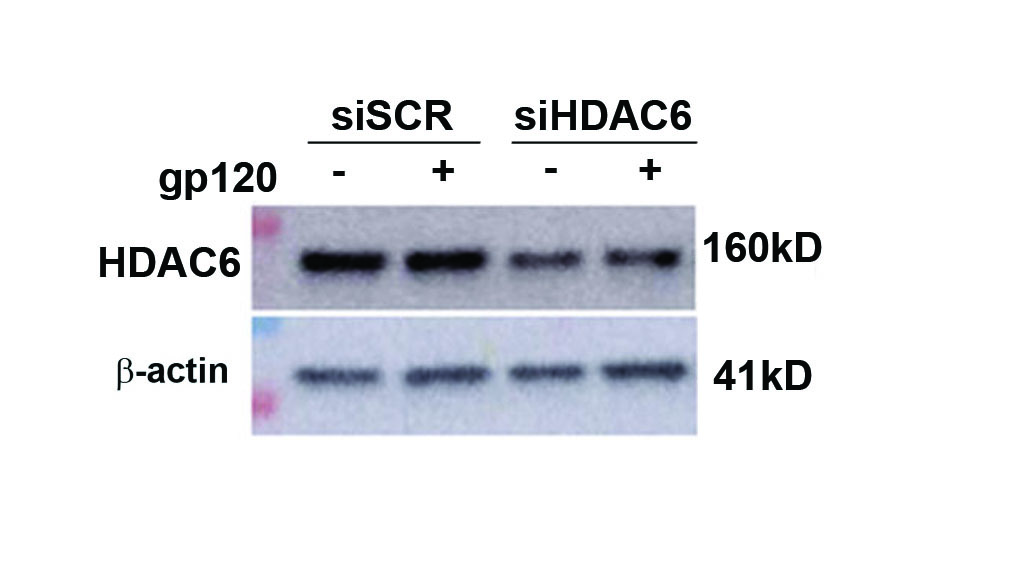

Supplement: Supplementary file 1 — Supplementary Figure 1 [file 41419_2019_1920_MOESM1_ESM.jpg]

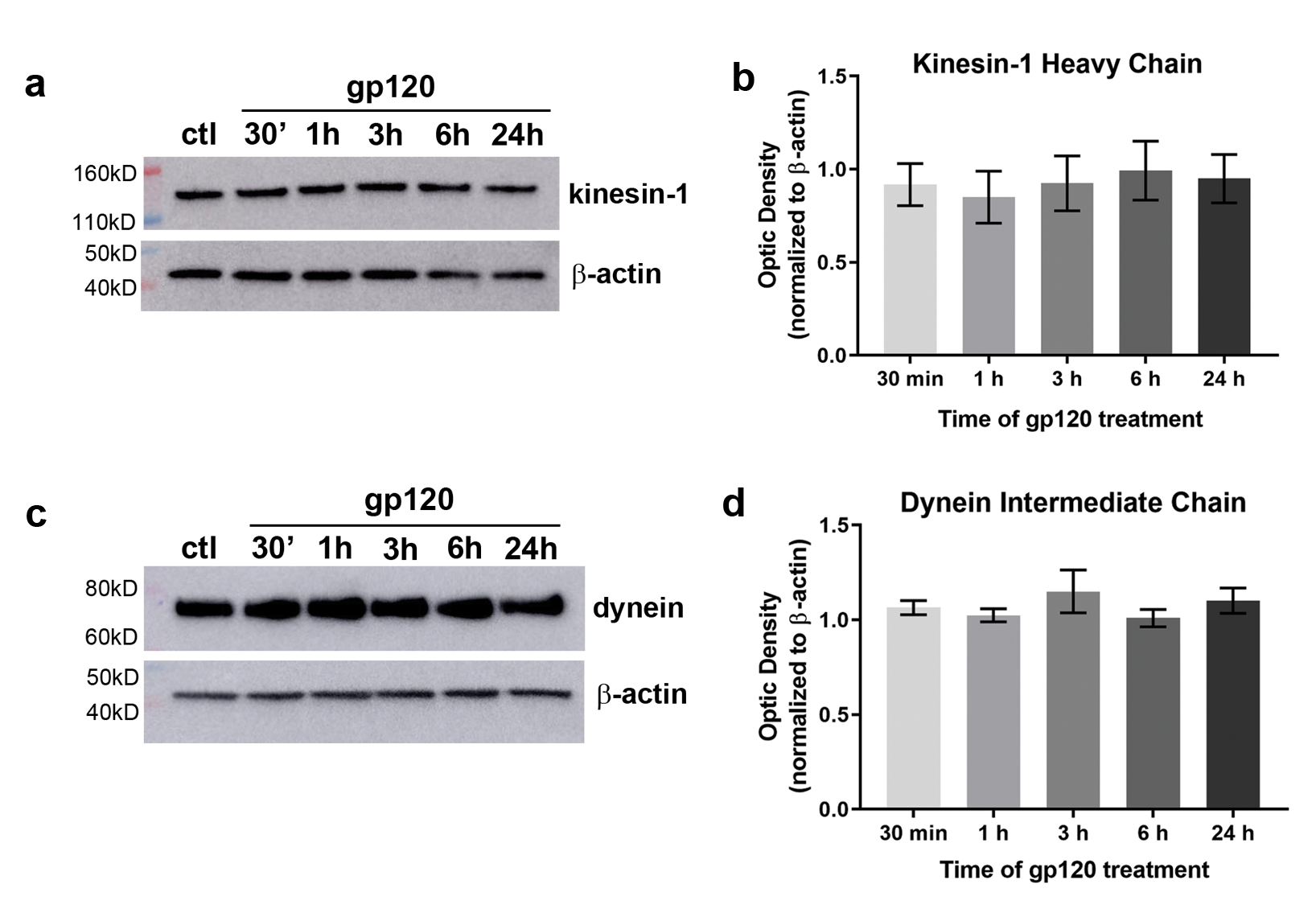

Supplement: Supplementary file 2 — Supplementary Figure 2 [file 41419_2019_1920_MOESM2_ESM.tif]

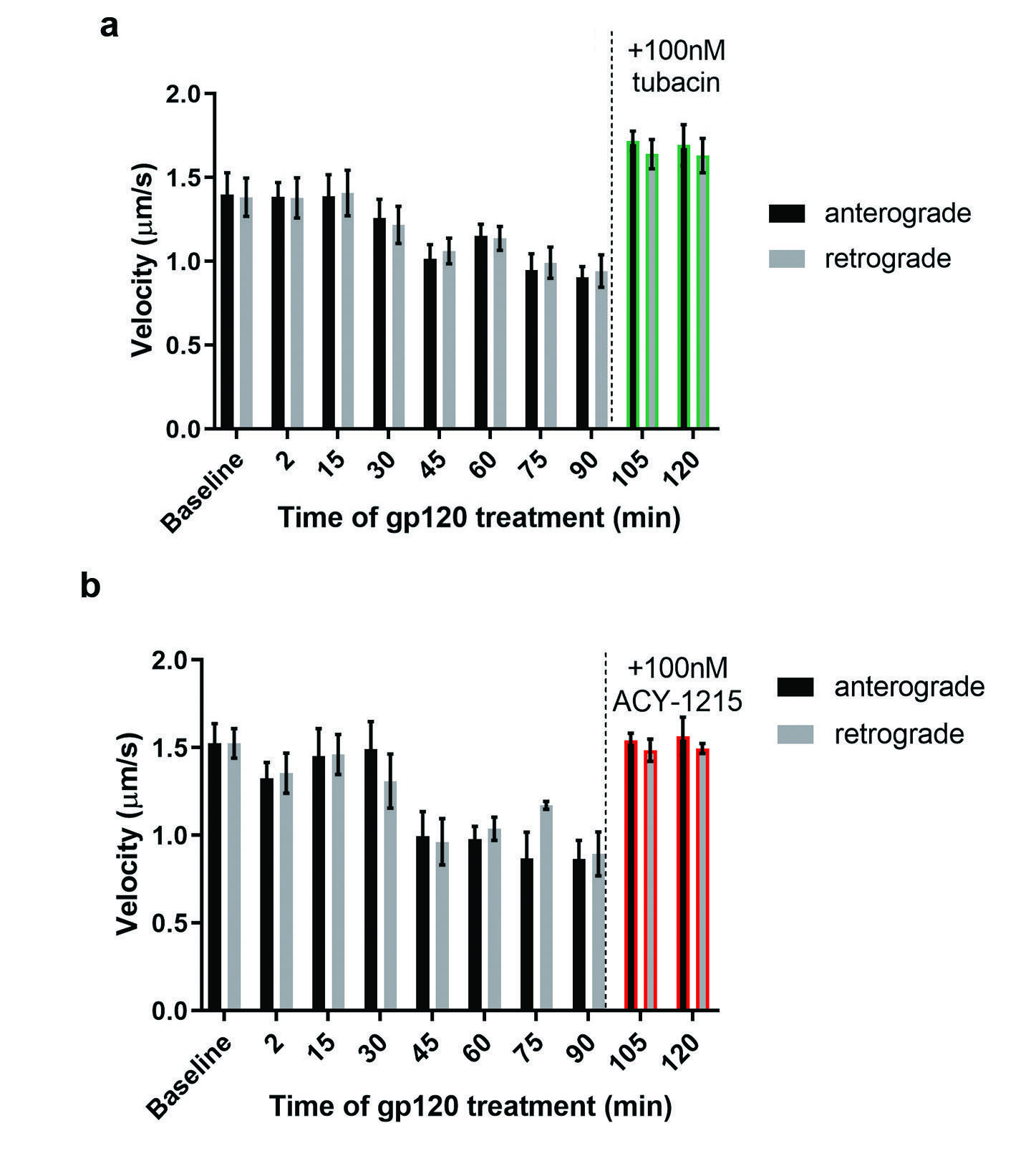

Supplement: Supplementary file 3 — Supplementary Figure 3 [file 41419_2019_1920_MOESM3_ESM.jpg]
